# Patient subgrouping with distinct survival rates via integration of multiomics data on a Grassmann manifold

**DOI:** 10.1186/s12911-022-01938-y

**Published:** 2022-07-23

**Authors:** Ali Alfatemi, Hong Peng, Wentao Rong, Bin Zhang, Hongmin Cai

**Affiliations:** Department of Computer Science and Engineering, South China University of Technology, Guangdong, China

**Keywords:** PCA, Grassmann manifold, Multi-omics data, Cancer subtypes, Patients subgroups, Survival rates

## Abstract

**Background:**

Patient subgroups are important for easily understanding a disease and for providing precise yet personalized treatment through multiple omics dataset integration. Multiomics datasets are produced daily. Thus, the fusion of heterogeneous big data into intrinsic structures is an urgent problem. Novel mathematical methods are needed to process these data in a straightforward way.

**Results:**

We developed a novel method for subgrouping patients with distinct survival rates via the integration of multiple omics datasets and by using principal component analysis to reduce the high data dimensionality. Then, we constructed similarity graphs for patients, merged the graphs in a subspace, and analyzed them on a Grassmann manifold. The proposed method could identify patient subgroups that had not been reported previously by selecting the most critical information during the merging at each level of the omics dataset. Our method was tested on empirical multiomics datasets from The Cancer Genome Atlas.

**Conclusion:**

Through the integration of microRNA, gene expression, and DNA methylation data, our method accurately identified patient subgroups and achieved superior performance compared with popular methods.

**Supplementary Information:**

The online version contains supplementary material available at 10.1186/s12911-022-01938-y.

## Introduction

The rapid development of high-throughput technologies has produced massive amounts of multiomics data, including genome, transcriptome, and proteome data as well as many more types. The analysis of these data enables researchers to improve basic research on cancer, including oncogene identification [1], recognition of cancer mutations [2], screening targets for cancer drugs [3], and cancer subtyping [4]. Many global projects, such as the International Cancer Genome Consortium (ICGC) and The Cancer Genome Atlas (TCGA), have also proven that multiomics data are invaluable for medicine. Thus, multiomics data integration is essential for understanding biological systems and distinguishing among different cancers.Moreover, fusing different datasets for a specific disease provides a more accurate comprehensive view of the disease, which facilitates diagnosis, treatment, guideline instructions, and prevention.

According to the manner of integration, methods for clustering multiomics data can be classified into three categories, namely, early, intermediate, and late integration [5, 6]. Early integration tends to rely on learning a common representation of the data, after which ordinary single-view clustering methods are implemented. In this strategy, the data are fused before the model is built; thus, the model ignores the connections among views [5, 7]. For late integration, the proposed model is applied to each type of data independently, and then integration is achieved by combining the results. Late integration methods lack a unified framework for integration, leading to unstable performance [8]. In contrast to the former two types, intermediate integration involves projecting multiomics data into an intermediate space or manifold and learning representations within this space for integration [7, 9, 10].

Inspired by the advantages of intermediate integration, we developed a novel integration method for handling high-dimensional multiomics data. We first learned low-dimensional representations for each view by utilizing principal component analysis (PCA). These representations can be regarded as new features of the patients within each view. With the obtained representations, we constructed a patient-to-patient graph for each view by using the k-nearest neighbors (k-NN) algorithm; these graphs served as intermediate representations, which we projected onto a Grassmann manifold. Finally, a new representation of the patients was obtained by merging these projections on the Grassmann manifold.

Its status as an intermediate integration method makes our method more advantageous than other methods based on early or late integration because it can accurately preserve the properties of each data type, thus making it a powerful approach for scaling different data. We tested our method by applying it to three types of data for many cancers, and we found it very suitable for multiple data that require integration for clustering. The method can be extended to include image datasets.

Our work focuses on the use of a well-known PCA technique based on Grassmann manifold theory that can be used to align different bases from different sources via a nonlinear alignment method. Nonlinear alignment methods, such as the Grassmann manifold method, can effectively improve the performance of clustering. Regarding multiomics data clustering, our work shows high superiority through the use of linking graphs and subspace theory.

The rest of this work is organized as follows. We first review recent progress in “Related works” section. Then, we present the methodology and results in “Methods” and “Results and discussion” sections, respectively. Finally, we present the conclusions in “Conclusion” section.

## Related works

The increasing significance of analyzing multiomics data has motivated many studies [11–13]. Facing the great difficulty of reducing the dimensionality of a dataset, the authors of [14] proposed a novel method of analyzing a breast cancer dataset using the PCA technique, which was used to capture the general structure of the clusters in the dataset. In the approach proposed in [15], PCA was utilized to find the topological structure among patients, and a previously unknown subgroup of breast cancers with 100% survival and no metastasis was elucidated.

To reduce the effects of bias and regular noise in heterogeneous genomic profiles, Shi et al. [16] proposed a pattern fusion analysis (PFA) framework that enables the identification of integrated sample patterns in a low-dimensional feature space. PFA obtains the local patterns of samples by synthesizing a specific feature space and a global sampling space across several types of datasets.

To further enhance the robustness of clustering [17, 18], the authors of [10] proposed a new method called similarity network fusion (SNF), in which patient similarity networks acquired separately from each omics dataset are integrated. The key step of SNF is to iteratively update the similarity of each view, and the final fusion is completed by averaging all similarity matrices. To avoid iterative optimization for SNF, in the GrassmannCluster method [7], patient-to-patient similarities are generated for multiple omics datasets and are mapped via subspace analysis on a Grassmann manifold.

## Methods

The proposed method consists of five steps, which are summarized as follows: (1) normalization, (2) dimensionality reduction, (3) construction of patient-to-patient graphs, (4) embedding of the k-NN graphs, and (5) merging on the Grassmann manifold.

### Normalization

Given *M* omics, let $X^{(m)}\in {\mathbb {R}}^{N\times D}$ denote the data matrices for omics *m*, where *N* and *D* are the numbers of patients and features, respectively. For all analyses, we perform the *z*-score transformation:

1 $$\begin{aligned} Z^{(m)}_{f,p}=\frac{X^{(m)}_{fp}- {\bar{X}}^{(m)}_f}{\sigma ^{(m)}_f } \end{aligned}$$

where $Z^{(m)}_{f,p}$ is the standardized value of feature *f* for patient *p* and ${\bar{X}}^{(m)}_f$ and $\sigma ^{(m)}_f$ are the mean and standard deviation, respectively, of feature *f*. Standardizing the expression values for each feature across all patients through *z*-score transformation [19] is necessary for running dimensionality reduction tools, such as PCA.

### Dimensionality reduction

The PCA technique has much wider applicability than other techniques, such as independent component analysis (ICA) and nonnegative matrix factorization (NMF). It is ideal for recognizing patterns and reducing dimensions; for more details, see the Additional file 1. As shown in previous references [20], the data after dimensionality reduction by PCA effectively capture significant patterns that are present in all the included datasets. After normalizing the data, we utilize PCA as a dimensionality reduction method to further extract important information from the data, and we select a sufficient number of components for each cancer type to explain up to 95% of the data variance.

We perform the PCA calculations as described in [20, 21]. Considering the *m*-th normalized matrix $Z^{(m)}$, the goal of PCA is to find the maximum projection variance of all samples, which can be formulated as:

2 $$\begin{aligned} \max _P Z^{(m)T}PP^T Z^{(m)}, ~s.t.~P^TP=I \end{aligned}$$

The matrix $P=[w_1, w_2, \ldots , w_k]$ is a standard orthogonal basis for a low dimensional space. The solution to Eq. (2) is made up of the top *k* eigenvectors of $Z^{(m)}Z^{(m)T}$. Suppose that $\lambda _1\ge \lambda _2\ge \cdots \ge 0$ are the eigenvalues of $Z^{(m)}Z^{(m)T}$ and that the associated eigenvectors are $w_1, w_2, \ldots , w_k$. Thus, the final result of PCA is calculated as $H^{(m)T} = P^TZ^{(m)}$.

### Construction of patient-to-patient graphs

We construct a patient-to-patient graph in the PCA space to model the specific structure within each view [22–24]. For the *m*-th graph $G^{(m)}=\{V^{(m)}, E^{(m)}\}$, the nodes $V^{(m)}$ denote patients within the space, and the edges represent the connections among these patients. . On this basis, we first compute the similarity matrix $W^{(m)}$ of graph $G^{(m)}$. Each element $W_{ij}^{(m)}$ measures the similarity between patients *i* and *j* and is computed as follows:

3 $$\begin{aligned} \begin{aligned} W_{ij}^{(m)}=e^{-\frac{ {\left\| H_{i}^{(m)}-H_{j}^{(m)} \right\| }^2 }{2t^{2}} },\quad i, j=1....N \end{aligned} \end{aligned}$$

The parameter *t* is the normalization factor [7]. The higher the value of this parameter is, the more similar the two patients are.

Next, we select the k-nearest neighbors of each patient to preserve the local structure of each graph:

4 $$\begin{aligned} \begin{aligned} {\widetilde{W}}_{ij}^{(m)} ={\left\{ \begin{array}{ll} W_{ij}^{(m)} &{}\quad if \,\, v_{i} \in {\mathcal {N}}_{i} \\ 0 &{}\quad \text{ otherwise } \end{array}\right. } \end{aligned} \end{aligned}$$

where ${\mathcal {N}}_i$ consists of the *k* nearest neighbors of patient *i*. The parameter *k* depends on the sample size. Different omics have distinct structures. Thus, the k-NN graph is more representative than the original similarity $W_{ij}^{(m)}$.

### Embedding of the k-NN graphs

To further extract crucial omics features, we project all the graphs into low-dimensional subspaces and obtain their associated embeddings in those spaces.

We first calculate the normalized graph Laplacian matrix $L^{(m)}$, which is defined as $L^{(m)}=D^{(m)-\frac{1}{2}}*(D^{(m)}-{\widetilde{W}}^{(m)})*D^{(m)-\frac{1}{2}}$, where $D^{(m)}$ is the degree matrix of the similarity ${\widetilde{W}}^{(m)}$, and each element is computed as $D_{ij}^{(m)}=\sum _{i}{\widetilde{W}}^{(m)}_{ij}$ [25].

With the learned Laplacian matrix, its embedding $U^{(m)}$ can be calculated by solving the associated eigenvalue problem according to the spectral clustering method:

5 $$\begin{aligned} \min \limits _{U^{(m)}\in R^{n*k}} tr(U^{(m)T}L^{(m)}U^{(m)}), \quad s.t. \quad U^{(m)^{T}}U^{(m)}=I \end{aligned}$$

The solution to Eq. (5) is the smallest *k* eigenvector of the normalized Laplacian matrix $L^{(m)}$. The embedding is the basis of each space. Thus, it is more comparable among omics than the original graphs.

### Merging on the Grassmann manifold

Minimizing the Euclidean distance between the integrated embeddings and the *M* embeddings of the omics is a natural way to obtain a fused representation:

6 $$\begin{aligned} \begin{aligned} \min \limits _{U\in R^{n*k}} \sum _{m=1}^{M}||U^{(m)} -U||^2_F,~s.t.\, U^{T}U=I \end{aligned} \end{aligned}$$

However, such a scheme assumes that similar patients are located close to each other in Euclidean space, which is often not the case. Multiomics data are complex and heterogeneous. Therefore, measuring their distance on a manifold, such as in a Grassmann manifold, rather than in Euclidean space is more appropriate.

The Grassmann manifold *G*(*k*, *n*) [26] is a set of *k*-dimensional linear subspaces. Each point on *G*(*k*, *n*) represents a set of orthonormal bases *Y* that can span a $k-$dimensional space *span*(*Y*). Thus, the distance between the spaces *span*(*Y*) and $span({\widetilde{Y}})$ can be defined as the sum of the principal angles for all the basis pairs:

7 $$\begin{aligned} d_{proj}^{2}(Y,{\widetilde{Y}})=\sum _{i=1}^{k} \sin ^2\Theta _{i}=k-tr(YY^{T}{\widetilde{Y}}{\widetilde{Y}}^{T}) \end{aligned}$$

where $\Theta _{i}$ is the principal angle between basis $Y_i$ and basis ${\widetilde{Y}}_i$ [26, 27].

Based on this measurement, the distance between subspace embeddings can be formulated as follows:

8 $$\begin{aligned}&\sum _{i=1}^{M}d_{proj}^{2}(U, U^{(m)}) \end{aligned}$$

9 $$\begin{aligned} =&kM-\sum _{i=1}^{M}tr(UU^{T}U^{(m)}U^{(m)T}) \end{aligned}$$

To minimize the discrepancy, we propose minimizing their geometric distance by minimizing the following objective function:

10 $$\min \limits _{U\in R^{n*k}} -\sum _{m=1}^{M}tr(UU^{T}U^{m}U^{m^{T}}),\quad s.t.\, U^{T}U=I $$

The Eq. (10) forces the integrated representation *U* to be close to all the embeddings $U^{(m)}$ in terms of the projected distance on the Grassmann manifold, and its solution consists of the *k* largest eigenvectors of the modified Laplacian matrix $L_{mod}=\sum _{m=1}^{M}U^{(m)}U^{(m)T}$. Finally, we cluster the results of subspace U integration by applying the k-means clustering algorithm.

## Results and discussion

In this section, we discuss the main results of our study. First, we introduce the datasets used in our work. Next, we explain the experimental procedures. Then, we compare our results with those of recent methods. Finally, we analyze the performance of all methods.

### Datasets

We used datasets processed using the SNF method, which were downloaded from the TCGA website and include data on breast invasive carcinoma (BIC), colon adenocarcinoma (COAD), kidney renal clear cell carcinoma (KRCCC), glioblastoma multiforme (GBM), and lung squamous cell carcinoma (LSCC). For each cancer type, three types of data are provided, namely, DNA methylation, gene expression, and miRNA expression. All these datasets contain stage III cancer data, as shown in Table 1.

**Table 1:** Patients (samples ) and features for the dataset

Cancer type (samples)	Gene expression(features)	DNA methylation (features)	MicroRNA (features)
BIC (105 patients)	17,814	23,094	354
GBM (215 patients)	12,042	1305	534
KRCCC (122 patients)	17,899	24,960	329
LSCC (106 patients)	12,042	23,074	352
COAD (92 patients)	17,814	23,088	312

### Experimental procedures

**Fig. 1:**
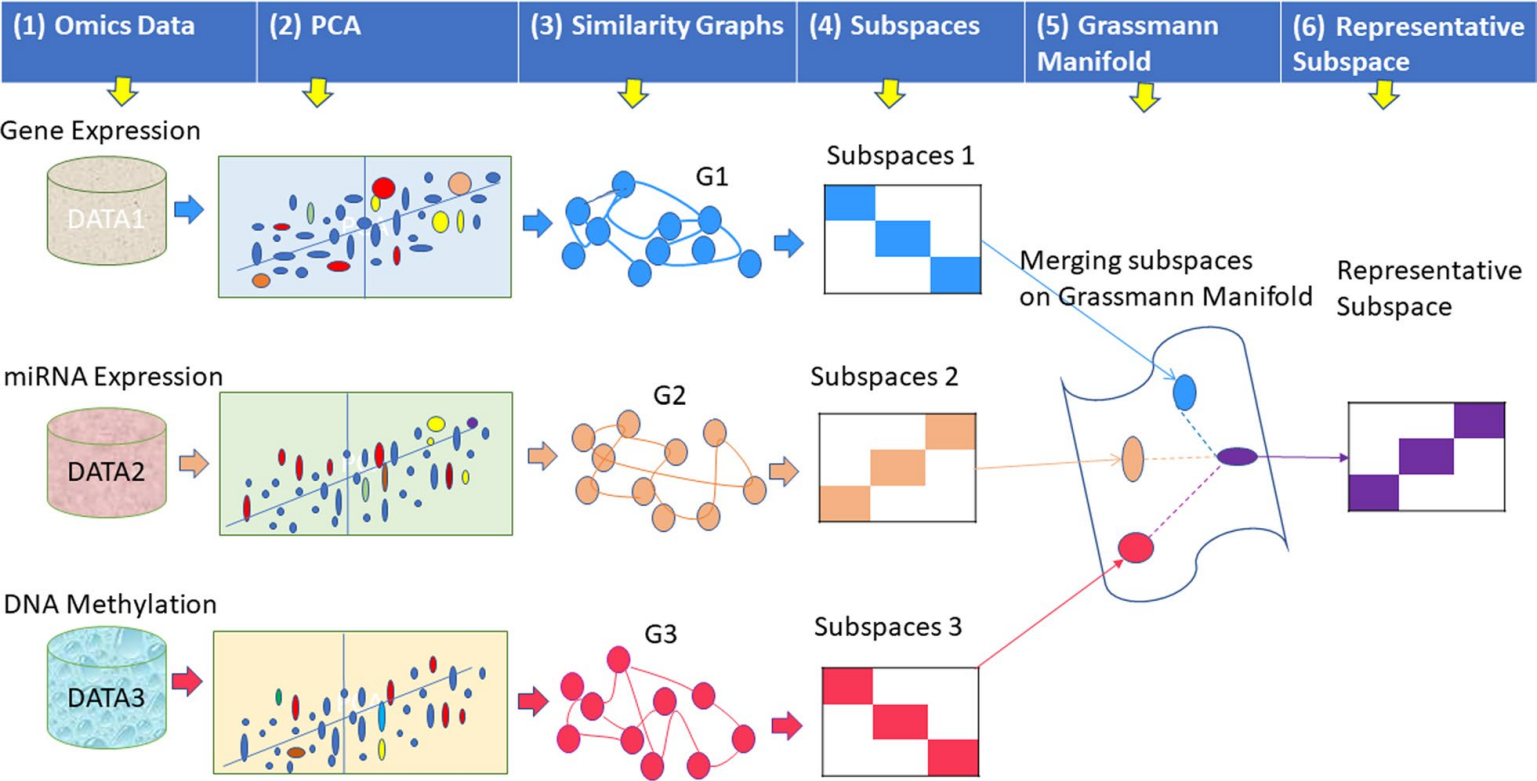
The framework of the proposed method. (1) The DNA methylation, gene expression, and miRNA expression omics datasets for the same cohort of patients. (2) The representation for each data type by using PCA. (3) The patient-to-patient graph for each type of omics data. (4) The subspace representation for graphs. (5) Subspaces merging via analysis on the Grassmann manifold. (6) The final integrative groups of patients

As shown in Fig. 1, we first preprocessed the omics data using z-score normalization. The original omics data have an enormous number of features. Therefore, in the proposed method, we utilized PCA to obtain reduced representations. Then, we created patient-to-patient graphs for each type of omics data. Note that we deleted edges with low similarity measures, which indicate uncertain relationships between samples. After obtaining all the graphs, we computed their *k*-dimensional spectral embeddings to further encode their information. Then, we merged all the representations on the Grassmann manifold. Finally, the patients were clustered in the fused representation, and we evaluated the clustering results via a post hoc analysis.

### Comparison with popular methods

To prove the efficiency of the proposed method, we compared it with SNF and the GrassmannCluster method. We used Cox survival *p* values to compare the results of our method with those of SNF and the GrassmannCluster method, and the results are shown in Table 2. For fair comparisons, we used the same number of group subtypes for each cancer for SNF and the GrassmannCluster method.

**Table 2:** Survival analysis by Log-rank test on five tumor dataset

Cancer type	GrassmannCluster	SNF	Our method
BIC (5 clusters)	$2.0\times 10^{-4}$	$1.1\times 10^{-3}$	$4.3\times 10^{-5}$
GBM (3 clusters)	$4.3\times 10^{-3}$	$2.0\times 10^{-4}$	$2.3\times 10^{-4}$
KRCCC (3 clusters)	$2.8\times 10^{-2}$	$2.9\times 10^{-2}$	$1.4\times 10^{-1}$
LSCC (4 clusters)	$1.6\times 10^{-2}$	$2.0\times 10^{-2}$	$2.7\times 10^{-3}$
COAD (3 clusters)	$4.2\times 10^{-2}$	$2.0\times 10^{-2}$	$2.7\times 10^{-3}$

Regarding the GrassmannCluster method, there was no COAD among the types of cancer. However, we used its code to obtain the Cox survival *p* values, as shown in Table 2. Four out of the 5 types of cancer obtained by the GrassmannCluster method were studied. Our method showed the important differences between the survival times. In SNF, 3 out of the 5 types of cancer were studied, showing that our method indicated significant differences in the survival times among subgroups.

Regarding the Grassmann clustering method, there was no COAD among the types of cancer. However, we used its code to obtain the Cox survival *p* values, as shown in Table 3. Four out of the 5 types of cancer were studied with the Grassmann clustering method. Our method showed important differences between the survival times. For SNF, 3 out of the 5 types of cancer were studied, and our method indicated significant differences in the survival times among subgroups.

**Fig. 2:**
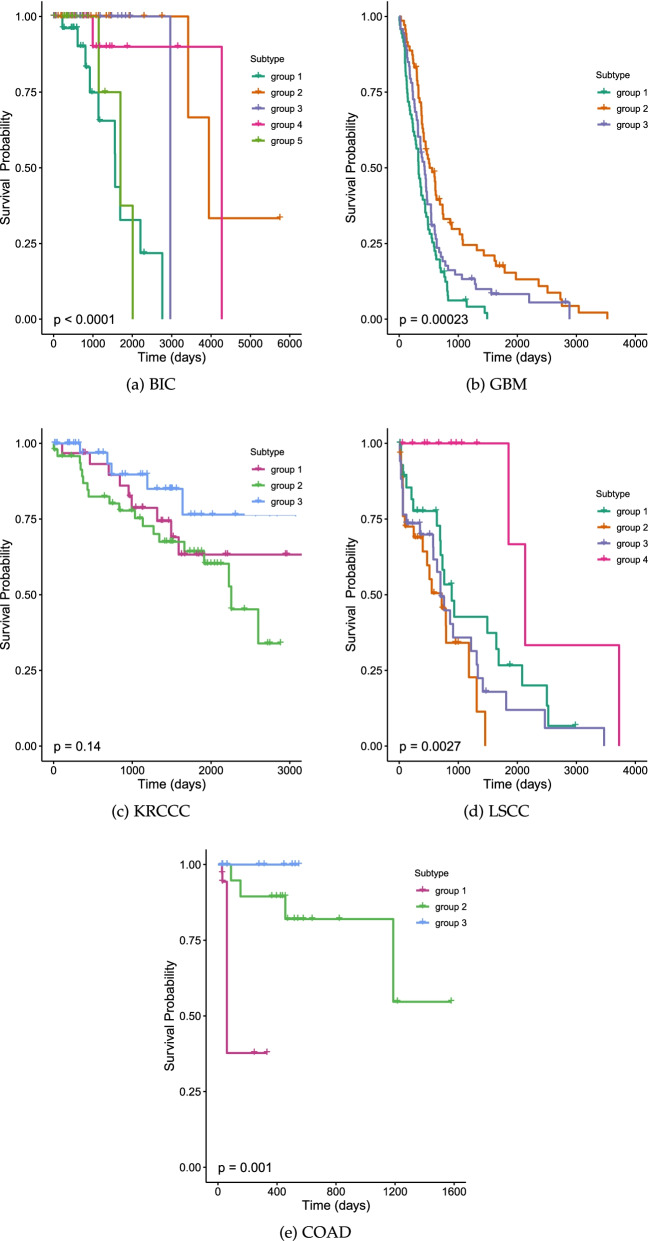
Performance comparison for generating subgroups for our method, SNF and GrassmannCluster using synthetic omics data

**Fig. 3:**
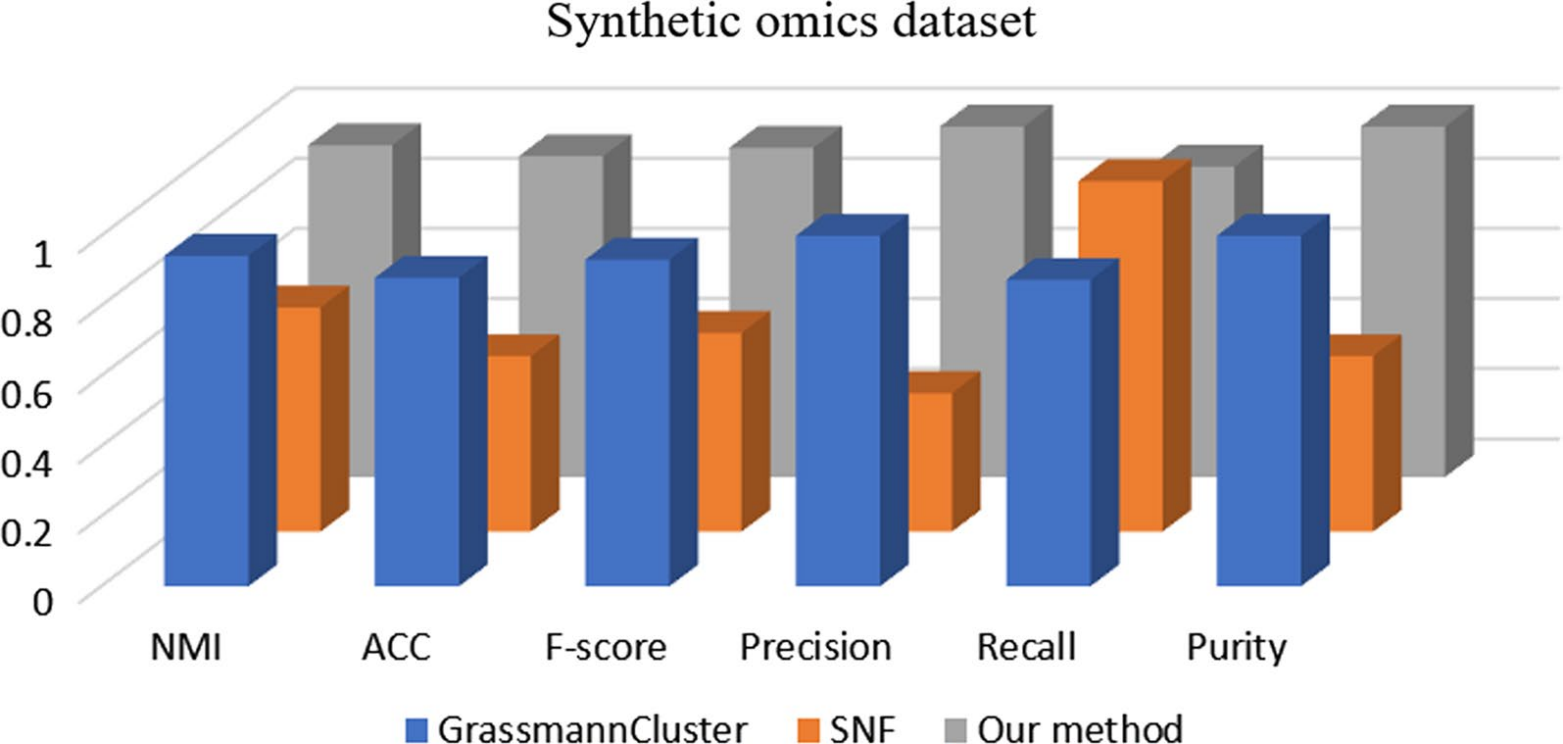
The clustering results heatmaps of similarities score for each type of cancer

**Table 3:** Clustering performance on synthetic multiple omics data. A higher value indicates better performance

Datasets	Methods	NMI	ACC	F-score	Precision	Recall	Purity
Synthetic omics data	GrassmannCluster	0.9429	0.8800	0.9320	1	0.8743	1
	SNF	0.639	0.500	0.5665	0.3952	1	0.5000
	Our method	0.9468	0.9150	0.9393	1	0.8855	1

Great care was taken to test a synthetic omics dataset extracted from [6, 28] to present comparisons. Out of 200 raw data samples, three types of data were simulated, namely, microRNA, gene expression, and DNA methylation data, which included 503, 2541, and 936 features, respectively. Then, we applied three mathematical clustering methods, that is, SNF, the GrassmannCluster method, and our method, to the synthetic omics dataset. As shown in Table 3, the performance was measured in terms of common quantitative measures: the accuracy (ACC), normalized mutual information (NMI), F-score, precision and purity. These metrics are widely used to evaluate clustering performance, with a higher value indicating better performance. The average results from ten runs are illustrated in Fig. 2. This figure shows that the proposed method outperforms the conventional methods in terms of accuracy. For example, when the SNF and GrassmannCluster methods are used, the accuracy is 0.500 and 0.8800, respectively, while the NMI is 0.639 and 0.9429; in contrast, our method results in an accuracy of 0.9150 and an NMI of 0.9468, both of which are better than the accuracy and NMI values achieved with the SNF and GrassmannCluster methods. In short, the results prove that our method is superior. The experiments were performed using a laptop computer with an Intel(R) Core(TM) i7-3537U CPU, 4 GB of RAM, the Windows 10 operating system, and MATLAB R2020A.

### Performance evaluation

To further demonstrate the superior performance of the proposed method, we plotted the heatmaps of all the similarities for each cancer type in Fig. 3. Breast cancer was selected as a case study, and the proposed method was applied to the TCGA data mentioned in “Datasets” section. The patients were clustered into five subgroups, as shown in Table 4. For more details, see Additional file 1: Section 6. The patients were grouped based on two main factors: (1) the silhouette score [29] to evaluate the most similar patients within the subgroups, for which it was found that k = 5 was the optimal value (see Additional file 1: Fig. S1 and Additional file 1: Fig. S2, and (2) the *p* value in the Cox log-rank test, to evaluate the significance of the differences in survival profiles between subgroups (see Additional file 1: Fig. S3).

**Table 4:** An example of survival to illustrate the comparison between five subgroups for breast cancer

	Subgroup 1	Subgroup 2	Subgroup 3	Subgroup 4	Subgroup 5
Number of patients	29	19	19	21	17
Events	10	2	1	2	3
Median (days)	1563	3945	2965	4273	1699
N.risk	5	2	1	1	2
Lower 95% CI	0.2188	0.0673	NA	NA	0.0839
Upper 95% CI	0.872	1	NA	NA	1
Survival	0.437	0.333	0	0	0.375

We compared the obtained subgroups against other known subgroups (luminal A, luminal B, triple-negative/basal-like, HER2-enriched and normal-like), for which we downloaded the data of already known subgroups from the TCGA website for the same patients. Kaplan–Meier and multivariate Cox analyses were performed to compare the known subgroups and the newly obtained subgroups. Significant survival differences were obtained for the newly obtained subgroups compared to the already known subgroups, as shown in Additional file 1: Fig. S4 and Table S1. It is clear that our obtained subgroups provide better survival percentages. Each group has a different survival effect, e.g., group 4 has the highest effect compared with the other groups, while group 1 has the worst survival prognosis (*P* value 0.001). Additionally, group 4 has the highest survival among all groups, with a hazard ratio (HR) of 0.02, a $95\%$ confidence interval (CI) of (0.001–0.3) and a *p* value of 0.008.

**Fig. 4:**
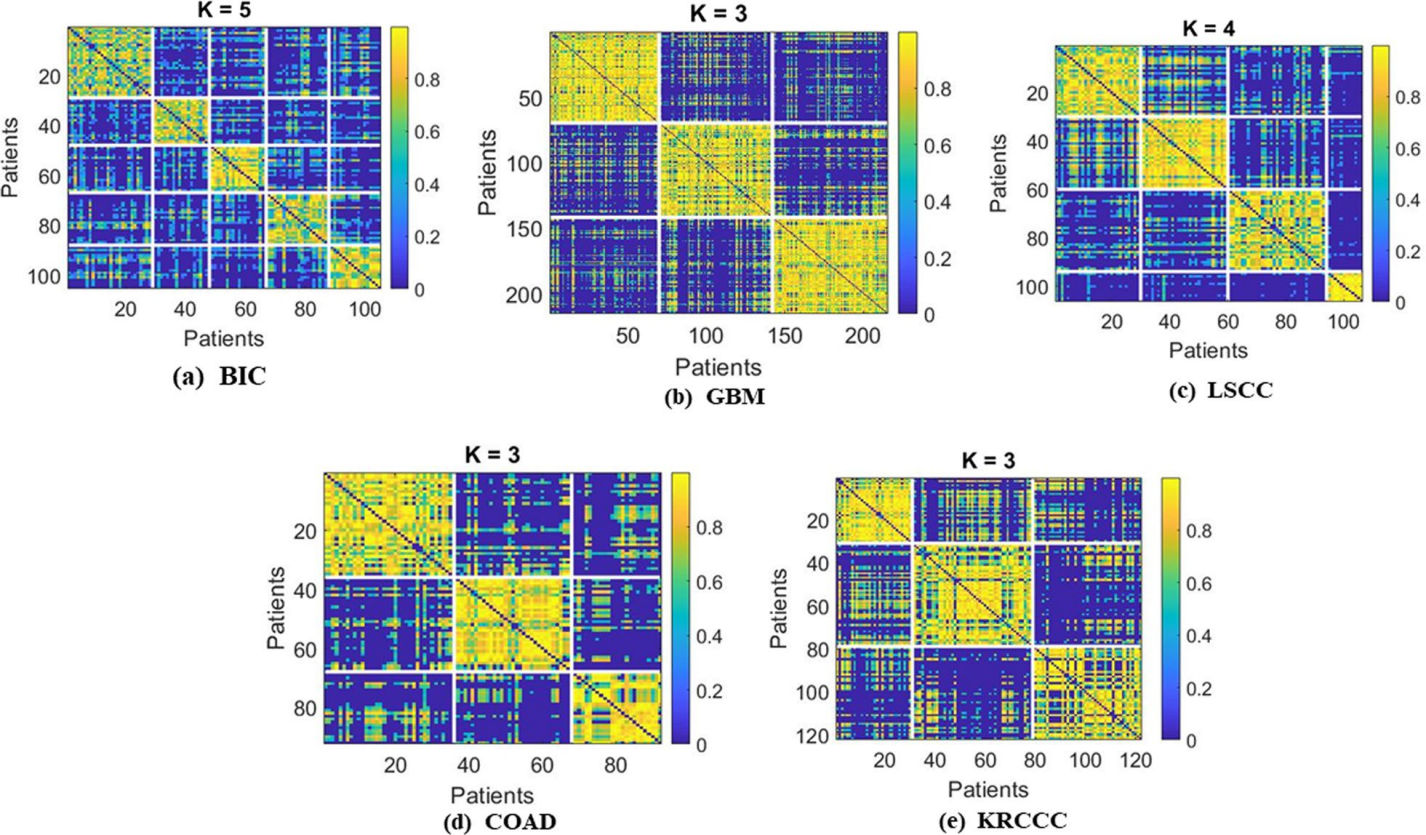
The integrative cluster detects clinically important subgroups of cancer, which are determined by Kaplan- Meier plots for survival clustering integration for BIC, COAD, GBM, KRCCC, and LSCC. The log-rank test show the *p* value by log-rank test summarizes the statistical differences between subgroups

In general, the differences in the survival profiles between subgroups were assessed by means of Cox log-rank tests using the survminer R package. The *p* value for breast cancer was $p<0.00001$ (see Table 2, Fig. 4). These results demonstrate that our method shows promising performance in cancer subgrouping. Consequently, we can conclude that our method provides superior and suitable performance in combining different types of omics data. Furthermore, our approach is very flexible in choosing suitable features for every patient.

## Conclusion

In this paper, we proposed a novel multiomics clustering method. Our method is based on subspace representation and manifold integration. We projected each type of omics data into a low-dimensional space via PCA and built a graph for the patients. Then, all the constructed graphs were represented by their spectral embeddings and subsequently merged on a Grassmann manifold. The proposed approach can effectively identify patient subgroups with distinct survival rates by combining microRNA, gene expression, and DNA methylation data. Our method was more accurate in addressing the patient subgroup problem than recent methods.

Our approach can be extended to include images, for which multiple types of properties need to be integrated. Moreover, our method can be used for other applications that require the integration of multiple types of features.

## Supplementary Information

**Additional file 1.** More details about using PCA, k-NN and k-means algorithms.

## Data Availability

TCGA (microRNA, gene expression, and DNA methylation ) datasets are available from Portal (https://portal.gdc.cancer.gov/) also it are publicly available in SNF method (http://compbio.cs.toronto.edu/SNF/SNF/Software.html). Programming language: Matlab R2020a and RStudio. The link to the code repository: https://github.com/ali20211/PCAG
