# Supplementary material for: Patient subgrouping with distinct survival rates via integration of multiomics data on a Grassmann manifold
Source: BMC Med Inform Decis Mak. 2022 Jul 23;22:190. doi: 10.1186/s12911-022-01938-y (PMC9308936; doi:10.1186/s12911-022-01938-y)
Supplement: Supplementary file 1 — Additional file 1. More details about using PCA, k-NN and k-means algorithms. [file 12911_2022_1938_MOESM1_ESM.docx]

**Patients subgrouping with distinct survival rates via integration of multiomics data on the Grassmann manifold**

Supplementary Information

Ali Alfatemi, Hong Peng, Wentao Rong, Bin Zhang and Hongmin Cai*

*Correspondence: hmcai@scut.edu.cn Department of Computer Science and Engineering, South China University of Technology, Guangdong, China

1. The incentive to use PCA

Since omics data have many features, an excellent technique for reduce the omics data dimensions is required. Therefore, dimensionality reduction is an essential step during clustering, especially in large datasets with large feature spaces. In this process, the number of random variables or features (attributes) is reduced. Several dimensionality reduction methods have been proposed. Principal component analysis (PCA) is a dimensionality reduction technique that reduces the number of variables without losing the information, and new variables with greater meaning are produced [1].

Moreover, an orthonormal and linear transformation is used by the PCA technique to combine correlated input variables into a set of linearly uncorrelated features known as principal components (PCs). PCA retains meaningful information by using correlations (covariances) and the variance of the raw variables [2],[3], [4]. We chose the PCA technique because it has much wider applicability than other techniques, such as independent component analysis (ICA) and nonnegative matrix factorization (NMF); it is ideal for recognizing patterns and reducing dimensions.

1. How PCA was employed in our method?

We ran our experiment on many single omics layers, such as microRNA, gene expression, and DNA methylation data, for five cancers types, namely, BIC, GBM, LSCC, COAD and KRCCC. For example, in breast cancer that contains gene expression, microRNA and DNA methylation data consist of 105 samples with different numbers of features between 353 and 23,095 features. We used the PCA function in MATLAB, [V, U] = PCA(single omics layer); we obtained two outputs [V, U], where V contains the loadings and U contains the score values. Then, we reconstructed the input data by U*V'. To perform dimensionality reduction, we must select the first n components of both matrices as U(:, 1:n) and V(:, 1:n) and perform the approximated reconstruction as U(:, 1:n)*V(:, 1:n)', where n is the number of components chosen to represent the data. We have chosen the number of components for each type of cancer that might allow explaining 95% of the data variance. For example, we used the first three PCA components in breast cancer, which explained 96% of the variance.

We utilized the PCA technique based on the Grassmann manifold with high superiority by linking graph and subspace theory. Our method achieved clustering's high performance to align the different bases from different sources via the nonlinear alignment method. As an example application of our method, we tested several multiomics datasets for many types of cancer, and we found that our method gives precise subtypes. Consequently, this example proves that our method works well. Our method is intermediate integration, and we used the most appropriate technique dimensionality reduction for data of the single omics layer as PCA according to many references such as Ranganathan, S., Nakai, K., Schonbach, C.: Encyclopedia of Bioinformatics and Computational Biology: ABC of Bioinformatics. Elsevier, (2018) [1].

1. How to choose the number of nearest neighbors?

The number of k in the k-NN algorithm normally depends on the sample size. We can indicate how to construct the graph and the number of nearest neighbors. We put an edge between two nodes if and only if they are among the k nearest neighbors of each other. We are required to provide the parameter k in the options; the default is k=30.

1. How can the number of clusters be chosen in the final k-means clustering step?

For the choice of the number of clusters in the k-means clustering algorithm, we used two methods in MATLAB to find an optimal k: evalclusters() and silhouette(). On the other hand, regarding the number of clusters in the k-means clustering algorithm, for the comparison between our method and the common methods mentioned in the results, we ensured that the division of cancer types in our study was identical to the traditional methods. On this basis, the number of clusters in the k-means clustering algorithm was selected, and we also ensured that it was an optimal k by using silhouette() in MATLAB as shown in figures 1,2. The k value is identical to the number of subtypes that we want, such as k = 5 for breast cancer. The second test for appropriate partition of the patients, was by the survival analysis Cox p-values and we found the fit partition when k=5 as shown in figure 3.


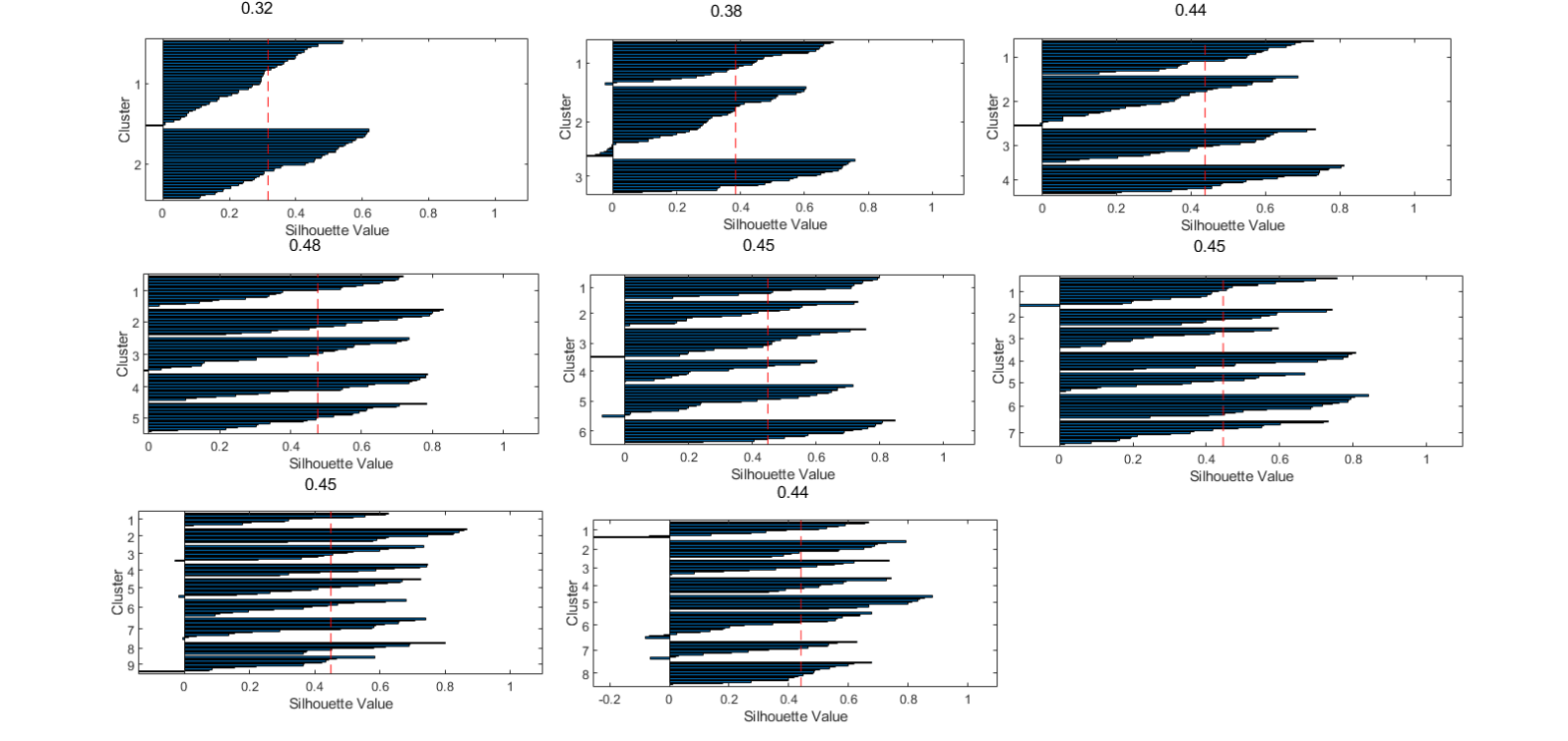


Fig. 1: Silhouette analysis for KMeans clustering


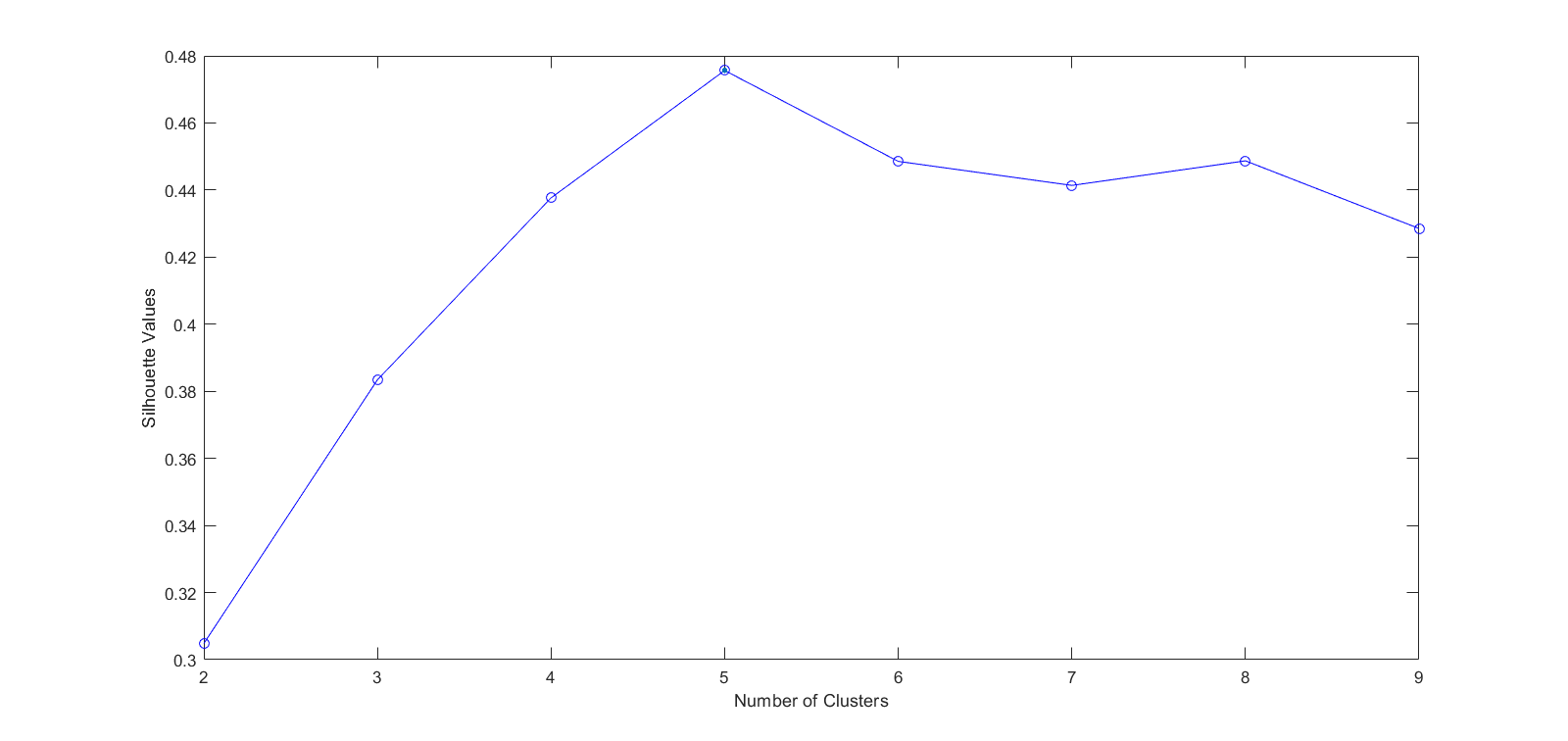


Fig. 2: The optimal K number of clusters, based on the silhouette criterion

| K=2 | K=3 |
| --- | --- |
| ***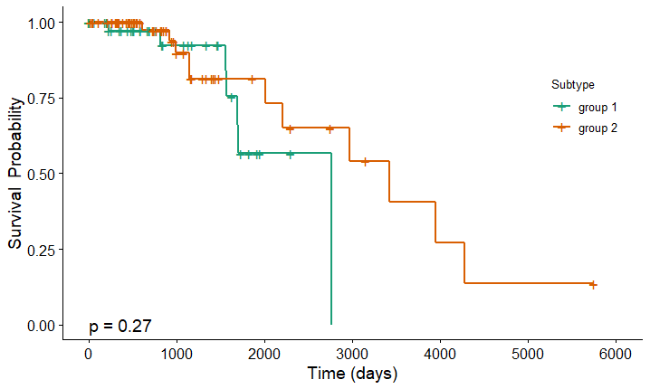*** | ***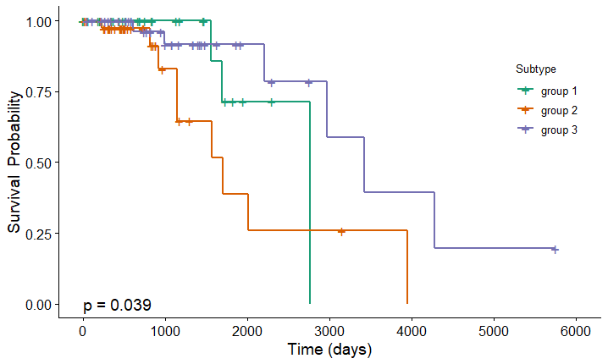*** |
| K=4 | K=5 |
| ***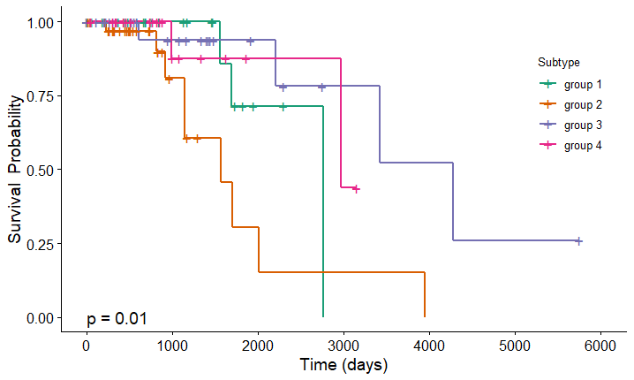*** | ***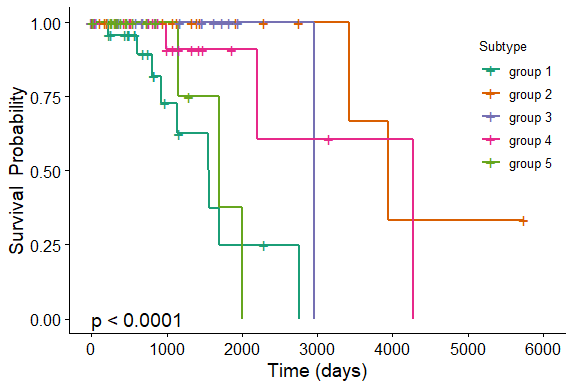*** |
| K=6 | K=7 |
| ***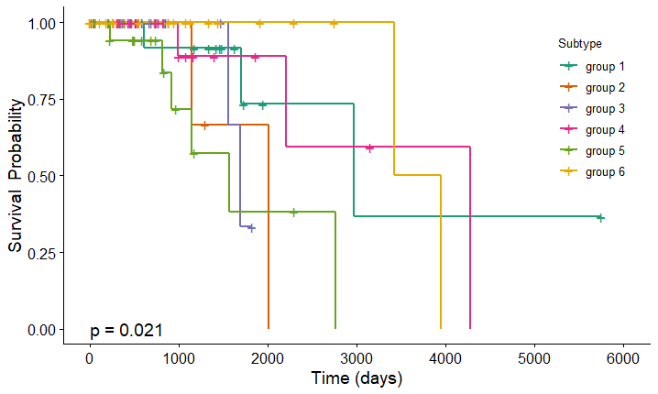*** | ***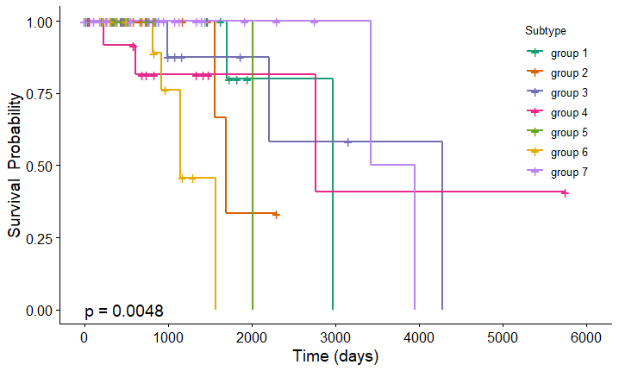*** |
| K=8 | K=9 |
| ***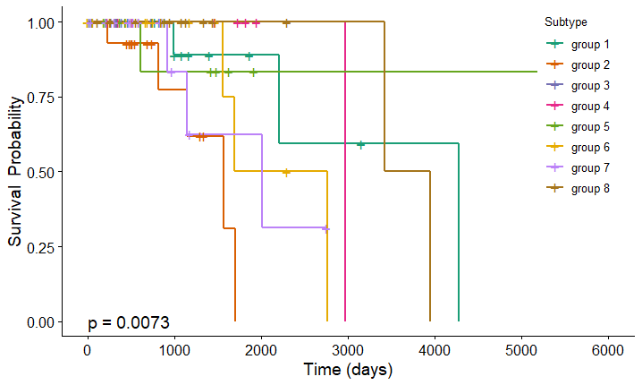*** | ***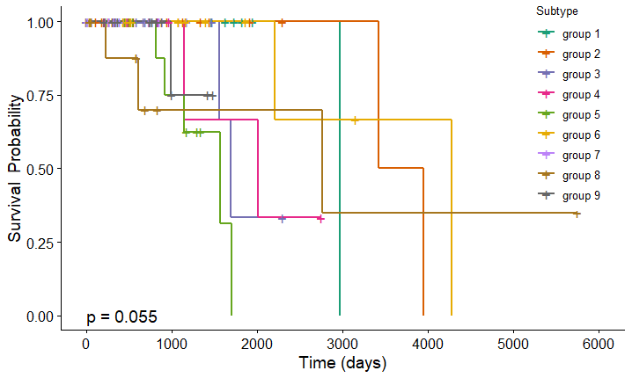*** |

Fig. 3: Appropriate partition test by the survival analysis Cox p-values

1. Synthetic data construction:

We are borrowed these data from HOPES method [5]. Synthetic data construction: A sample process of creating simulated data is depicted. Borrowed code first have the actual genomic data from RNA expression, DNA methylation, and miRNA expression in the first column. Then it creates three distinct matrices with various clusters in the second column. The simulated data were obtained by the combination of the first and the second column, respectively. The actual genomic profiles involved researches on DNA methylation, RNA expression and miRNA expression were downloaded from GEO (https://www.ncbi.nlm.nih.gov/geo/) with the following GEO codes: GSE51557, GSE73002 and GSE106453. For each data type, a missing-value imputation was performed to preprocess the data. 200 samples with a missing rate lower than 20% were randomly selected. For those having missing-value data, a K-nearest neighbor (KNN) imputation scheme was used to complement it by filling the empty area with the mean value of non-empty neighbors from the 200 complete samples. for more information for each matrix, please see (Supplementary Material: Simultaneous interrogation of cancer omics to identify subtypes with significant clinically differences, <https://www.frontiersin.org/articles/10.3389/fgene.2019.00236/full#supplementary-material>).

1. Details about molecular subgroups of breast cancer

We divided breast cancer into five groups. For subtype 1, we observed that 10 events occurred out of 29 patients. Among the individuals who survived to the median survival time was 1563 days, the event (death) occurred for five people. The ratio of survival was 0.437 with a 95% confidence interval of (0.2188-0.872), where events are the number of deaths and the ratio of survival was observed through Kaplan-Meier estimation. n.risk is the number of subjects at risk at a specific time.

For subtype 2, we observed that two events occurred out of 19 patients. When the median survival time was 3945 days in group 2, the risk occurred for two people who may die or relapse, and the ratio of survival was 0.333 with a 95% confidence interval of (0.0673-1) for the proportion of individuals who survived to that point. For subtype 3, we observed that one event occurred in 19 patients. The median survival time was 2965 days for subtype 3, the risk occurred for one person who may die or relapse. The ratio of survival was 0 days, indicating that there was no case for censoring. Moreover, for subtype 4, we observed that two events occurred out of 21 patients. The median survival time was 4273 days in group 4, the risk occurred for one person who may die or relapse. The ratio of survival was 0 days, indicating that there was no case for censoring. Last, for subtype 5, we observed that three events occurred in 17 patients. The median survival time was 1699 days in group 5, the risk occurred for two people who may die or relapse, and the ratio of survival was 0.375 with a 95% confidence interval of (0.0839-1) for the proportion of individuals who survived to that point.

Table 1: Show the significance for new subgroups and already know subgroups using multivariate cox analysis

| Subgroup | subgroups already known | New subgroup |
| --- | --- | --- |
| Subgroup 1 (Luminal A) | 0.212 | 0.003 |
| Subgroup 2 ( HER 2) | 0.439 | 0.001 |
| Subgroup 3 (basal) | 0.318 | 0.01 |
| Subgroup 4 (Normal) | 0.315 | 0.012 |
| Subgroup 5 (Luminal B) | 0.058 | 0.226 |

Fig. 4: The comparison between already know subgroups and new subgroups using Cox regression


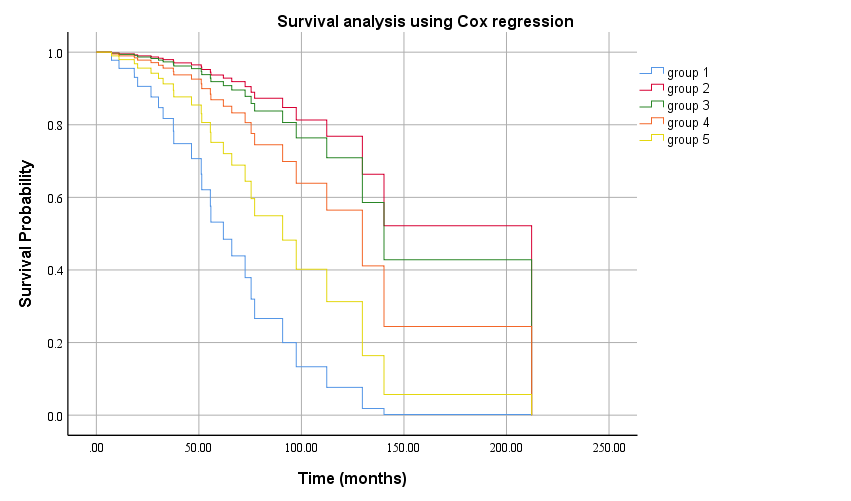

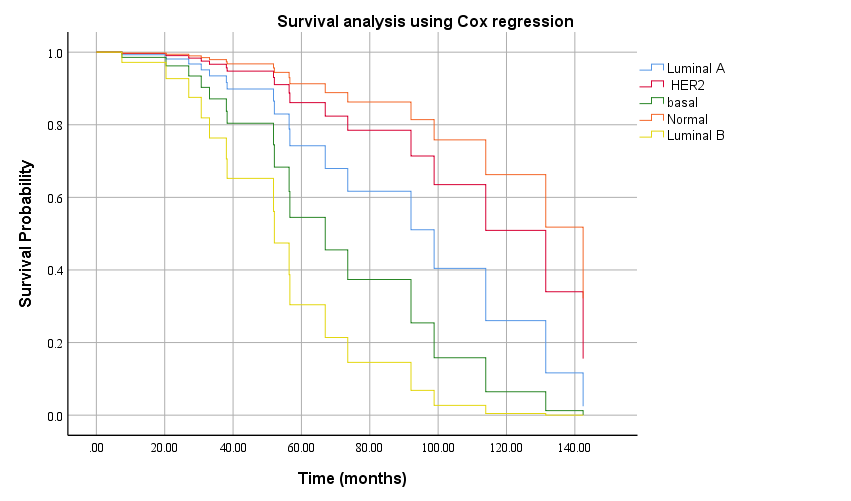


## References

[1] S. Peter *et al.*, “Encyclopedia of Bioinformatics and Computational Biology,” *researchgate.net*.

[2] I. T. Jolliffe and J. Cadima, “Principal component analysis: a review and recent developments,” *royalsocietypublishing.org*, vol. 374, no. 2065, Apr. 2016, doi: 10.1098/rsta.2015.0202.

[3] M. Ringnér, “What is principal component analysis?,” *Nature Biotechnology*, vol. 26, no. 3. Nature Publishing Group, pp. 303–304, Mar. 2008, doi: 10.1038/nbt0308-303.

[4] J. S. preprint arXiv:1404.1100 and undefined 2014, “A tutorial on principal component analysis,” *arxiv.org*.

[5] A. Xu, J. Chen, H. Peng, G. Q. Han, and H. Cai, “Simultaneous interrogation of cancer omics to identify subtypes with significant clinical differences,” *Front. Genet.*, vol. 10, no. MAR, 2019, doi: 10.3389/fgene.2019.00236.
